# Immediate outcome of endovascular treatment of ruptured juxtarenal aneurysm with parallel stents

**DOI:** 10.1590/1677-5449.200120

**Published:** 2021-03-15

**Authors:** Claudia Guimarães Agle, César Amorim Pacheco Neves, Flávia Dórea Carneiro Abbehusen, Tainã Lemos Andrade, Filinto Marques de Cerqueira, Dejean Sampaio Amorim

**Affiliations:** 1 Centro Universitário FTC – UniFTC, Salvador, BA, Brasil.; 2 Associação Bahiana de Medicina – ABM, Salvador, BA, Brasil.; 3 Colégio Brasileiro de Radiologia – CBR, São Paulo, SP, Brasil.; 4 Fundação Bahiana de Cardiologia – FBC, Salvador, BA, Brasil.

**Keywords:** ruptured aneurysm, endovascular procedures, abdominal aortic aneurysm, stents, rupture

## Abstract

Rupture of an abdominal aortic aneurysm is an event with a high mortality rate and treatment is a medical emergency. Endovascular treatment of these aneurysms has become established as a minimally invasive alternative to classical open surgery and is now the first-choice option. However, 20 to 50% of patients with abdominal aortic aneurysms do not have anatomy favorable for endovascular treatment because of a short aneurysm neck or because visceral branches are involved by the aneurysm. We report the case of a 70-year-old patient who underwent endovascular repair of a ruptured juxtarenal aneurysm with deployment of parallel stents in the renal arteries (in a chimney technique). Clinical data and details of the procedure are reported. Technical success was achieved and there were no postoperative complications.

## INTRODUCTION

Abdominal aortic aneurysms (AAA) are a challenge for vascular surgeons, especially when they extend to the visceral vessels (complex aneurysms). Rupture of an AAA is an event with high mortality and surgical or endovascular treatment of these cases is a medical emergency. The risk of rupture of an aneurysm is proportional to its size and aneurysms measuring less than 5.4 cm have an annual rate of rupture of approximately 1%, while those exceeding 7.0 cm in diameter have an annual rate of rupture of 32.5%.^1^ This risk is four times greater in women than in men.^2^ Endovascular treatment (ET) of these aneurysms has become established as a minimally invasive alternative to classical open surgery and is now the first-choice option. This progress is primarily based on the multicenter randomized trials EVAR-1 (Endovascular Aneurysm Repair 1), DREAM (Diabetes Reduction Assessment with ramipril and rosiglitazone Medication), and OVER (Open vs. Endovascular Repair).^3^^-^ 6 However, 20 to 50% of patients with AAA do not have favorable anatomy for ET, because of a short neck or because visceral branches are involved by the aneurysm, and it is known that AAAs with proximal neck lengths shorter than 10 millimeters are associated with increased risk of reintervention and death.^7^ Feasible options for these cases include techniques using with fenestrated stents, branched stents, surgeon-modified stents, or parallel stents. The parallel stents (chimney) technique is based on placing stents into the visceral vessels in parallel with the main endograft body.^8^ Here, we present a case of rupture of a juxtarenal AAA that was treated at a private center in the city of Salvador, BA, Brazil, using endovascular techniques to implant parallel stents for the renal arteries.

The study was approved by the Ethics Committee at the Fundação Bahiana de Cardiologia (FBC) in Salvador, BA, Brazil (Decision number 4.341.672).

## PART I - CLINICAL SITUATION

**Figure 1:**
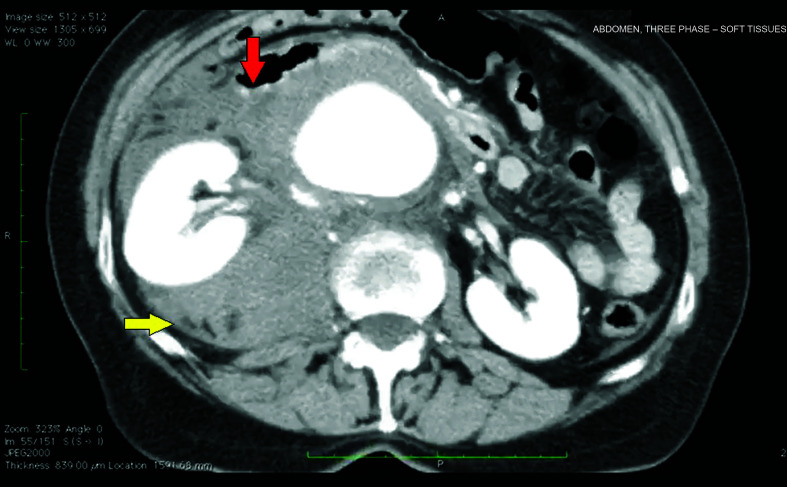
Angiotomography showing a ruptured abdominal aortic aneurysm (AAA) with right-side retroperitoneal hematoma. Red arrow: aneurysm rupture (AAA wall broken). Yellow arrow: retroperitoneal hematoma

**Figure 2:**
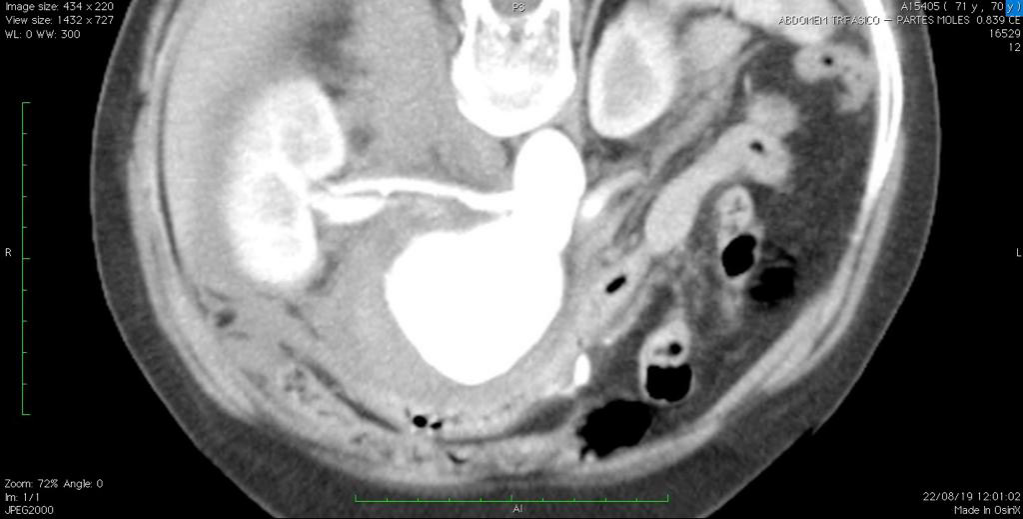
Angiotomography showing juxtarenal abdominal aortic aneurysm.

**Figure 3:**
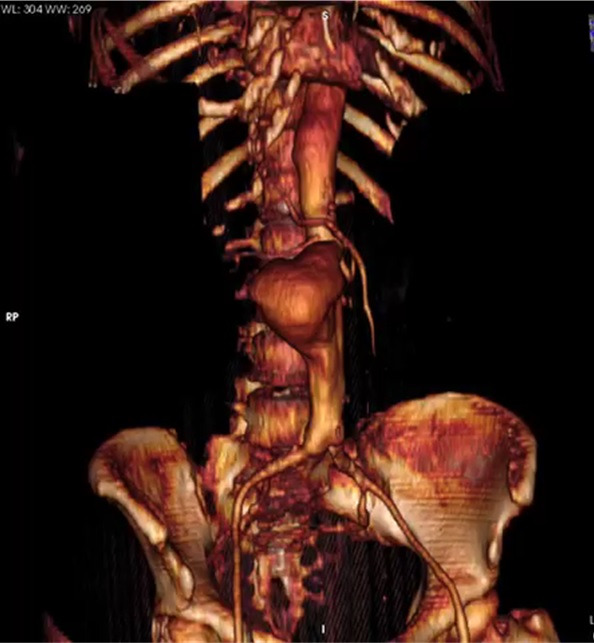
Tomographic reconstruction showing juxtarenal abdominal aortic aneurysm.

The patient was a 70-year-old female smoker with hypertension. She had been referred to the service with intense pain in the right flank and the pelvis. Investigative work-up proceeded on the basis of a diagnostic suspicion of nephrolithiasis and tomography with contrast was ordered. This examination detected an 8cm fusiform aneurysm with signs suggestive of rupture (Figure 1) and juxtarenal position (Figures 2 and 3).

In view of this presentation, the following treatment options were considered:

1- Endovascular procedure (fenestrated stent, branched stent, surgeon-modified stent, or a parallel stenting technique);
2- Conventional open surgery.

## PART II - WHAT WAS DONE

**Figure 4:**
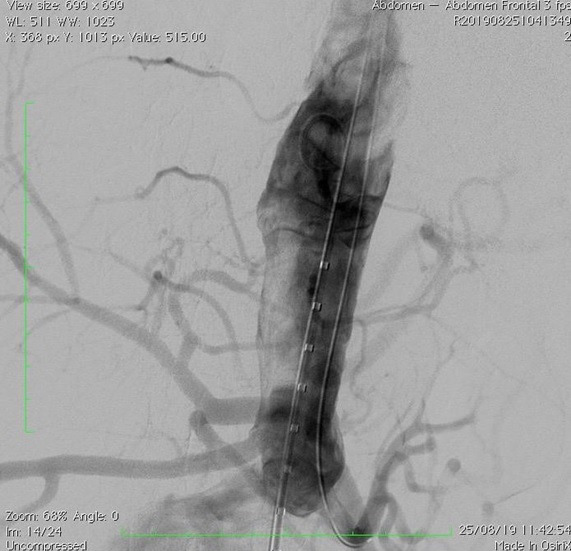
Endoprosthesis body positioned in the proximal aorta, with selective catheterization of the left renal artery.

**Figure 5:**
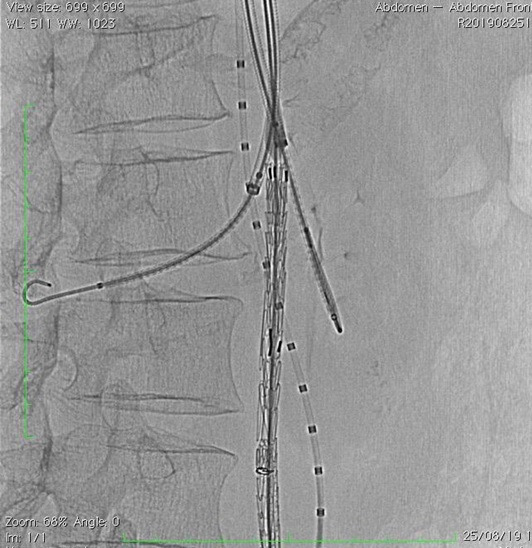
VIABAHN self-expanding stents positioned in the renal arteries.

**Figure 6:**
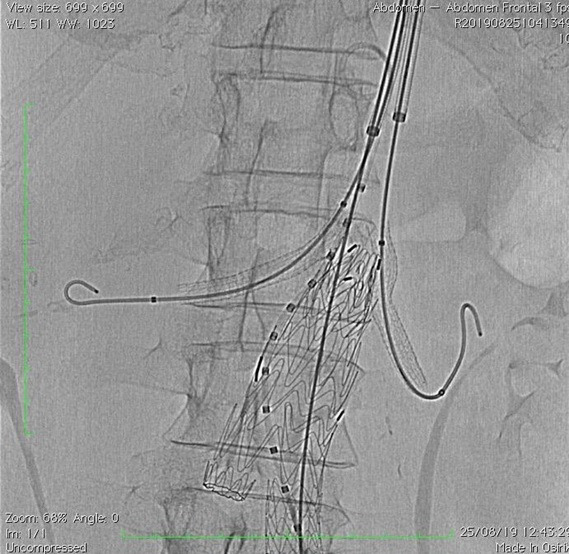
Endoprosthesis and self-expanding renal stents released.

**Figure 7:**
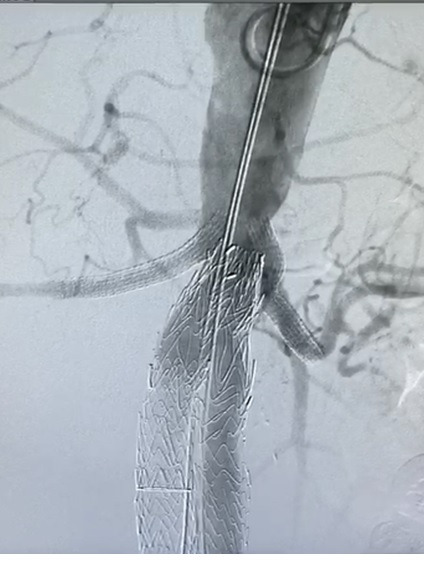
Control aortography showing proximal segment of the endoprosthesis free from leaks and stents correctly positioned in the renal arteries.

**Figure 8:**
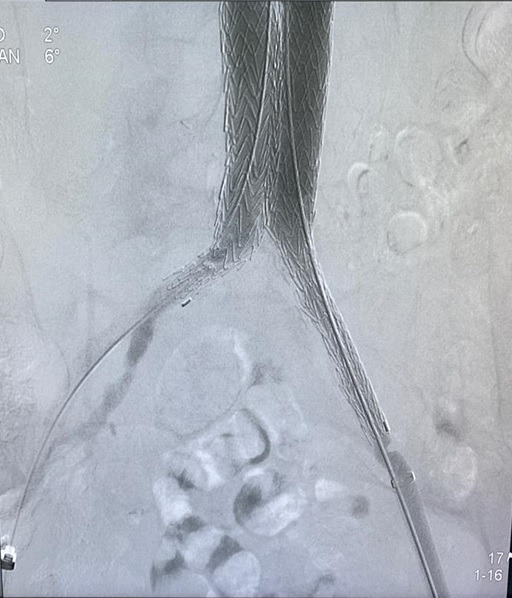
Control aortography showing distal segment of the endoprosthesis free from leaks.

**Figure 9:**
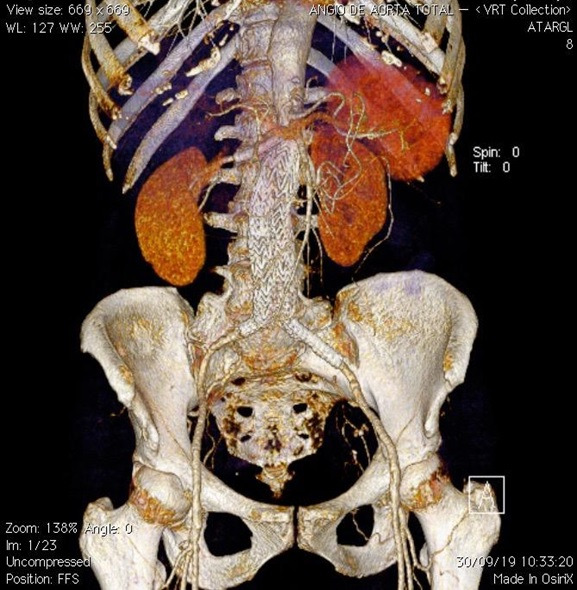
30-day control angiotomography showing endoprosthesis and renal artery stents correctly positioned and free from fractures or leaks.

The patient underwent general anesthesia and endovascular treatment was conducted. Four accesses were needed for the procedure: bilateral dissection of the common femoral arteries and puncture of both brachial arteries. A 12Fr introducer was inserted into the right femoral artery, a 16Fr introducer into the left femoral artery, and long 7Fr introducers into the brachial arteries. The left femoral artery access was used to position the main endoprosthesis body (Figure 4) GORE C3^®^ (W. L. Gore & Associates, Inc., Delaware, United States) (26 x 14 x 18) close to the superior mesenteric artery. The brachial arteries were used to catheterize the renal arteries and position self-expanding covered stents VIABAHN 6 x 50 (Figure 5). The endoprosthesis body was released up to the contralateral leg, and then the renal artery stents were released (Figure 6). The right femoral artery was then used to deploy the contralateral extension of the endoprosthesis (16 x 14 x 12). Control aortography showed the endoprosthesis were leak free and the renal arteries were patent (Figures 7 and 8). A balloon was not used to fit the proximal segment of the endoprosthesis. The patient stayed in the intensive care unit (ICU) for 24 hours and was discharged from hospital after 48 hours. Control tomography at 30 days showed that the endoprosthesis and the stents in the renal arteries were well-positioned and free from signs of fracture (Figure 9).

## DISCUSSION

The time to intervention has a direct impact on the results of treatment of a ruptured AAA. These patients’ diagnosis must be identified as early as possible, with rapid referral to a hospital that has the infrastructure needed for adequate treatment of the pathology. According to the 2018 Society for Vascular Surgery (SVS) practice guidelines, the target door-to-balloon time should be less than 90 minutes, with time zero defined as the first medical contact, and intervention defined as initial arterial access and deployment of an aortic occlusion balloon. However, while this is the recommendation, it is still a challenge to provide care in this short time frame. In 2004, the United States’ National Cardiovascular Data Registry stated that just 8% patients who needed inter-hospital transfer achieved the door-to-balloon time of less than 90 minutes, with a mean time of 152 minutes.^9^

Currently, ET of ruptured AAA is reserved for stable patients, because it is necessary to conduct angiotomography in advance to define the most appropriate measures for this type of intervention. However, high-demand referral centers have protocols in place for cases that tend to benefit from resuscitative endovascular balloon occlusion of the aorta, which extends the applications of ET for unstable patients.^10^ It should be remembered that this conduct is not consensus, is still rarely discussed in Brazil, and was not used in our case.

A trend analysis conducted in the United States confirmed that EVAR is increasingly being used to treat ruptured AAA and that the trend is associated with reduced mortality. The analysis also showed that results are better when EVAR for ruptured aneurysms is performed at teaching hospitals and high-volume centers. When anatomically favorable, the option for ET rather than open surgery was classified as having a strong recommendation level (level 1).^11^ In Brazil, the Brazilian Guidelines for AAA Treatment, published by the Brazilian National Health Service’s (SUS - Sistema Único de Saúde) National Commission for Technology Adoption (CONITEC), state that stable ruptured AAAs with anatomy favorable for treatment with EVAR (confirmed with computed tomography) should be treated on an emergency basis with open surgery or EVAR, depending on the experience of the surgical team and availability of the materials needed.^2^

The randomized multicenter IMPROVE trial (Immediate Management of Patients with Ruptured Aneurysm: Open Vs. Endovascular Repair) demonstrated similar 30-day mortality for open repair (37.4%) and endovascular repair (EVAR) (35.4%). However, EVAR had a shorter length of hospital stay. There is also a trend for better results in women.^12^

The endoprostheses account for a significant proportion of the cost of EVAR (34-52%), but this expenditure is compensated by the shorter length of hospital stay, which differentiates EVAR from open repair. However, significant differences in cost are not observed over the long term because of the need for follow-up with imaging exams and reinterventions after EVAR. Patients treated with EVAR generally have better health-related quality of life during the first 12 months, although studies have not shown significant differences after the first year.^13^

Benefits of the endovascular procedure compared to open repair include its reduced invasivity, avoiding laparotomy, vascular control under local anesthesia, hemodynamic stability, permissive hypotension during the entire procedure, and selective aortic occlusion to reduce retroperitoneal hemorrhage. This can result in a shorter duration operation, less blood loss, and reduced perioperative cardiopulmonary morbidity and mortality, possibly resulting in better 30-day mortality outcomes and better long-term outcomes.^14^ However, it is important to be alert to the size of the aneurysm to be repaired, because it has been reported that the larger the diameter of the AAA, primarily observed in cases with rupture, the stronger the association with reintervention rates.^15^

The parallel stenting technique consists of placing covered stents in the visceral arteries, in parallel with the body of the aortic endoprosthesis. The method is known as a periscope technique when placed retrograde to the direction of blood flow and as chimney, snorkel, or sandwich techniques when placed in the direction antegrade to flow. Normally, for ET of AAA, 10 to 15% endoprosthesis oversizing is used. However, with the chimney technique, greater oversizing (30%) is needed to avoid the possibility of gutter leaks. It is also important to ensure an overlap (length) of at least 5 cm of the parallel stent, ending 1 cm above the aortic endoprosthesis.^16^ Ballooning should not be used to fit the graft to the aorta because of risk of compression of the parallel visceral stents.

The decision to choose the chimney technique was made for several reasons: easy access to the materials needed for ET, a team with experience in the technique, and the possibility of providing treatment in a short period. Additionally, use of a fenestrated stent was restricted by the time taken for manufacture (6-8 weeks), since they must be custom-made for each case. Branched endoprostheses could not be used because of the need for a minimum diameter of 34 mm. In turn, off-the-shelf endoprostheses are still only available on the market with four branches or fenestrations (which was unsuitable for this case in which there was only a need to cover the renal arteries).^17^

Finally, with regard to surgeon-modified endoprostheses, they demand greater experience with handling and modification of the devices. There is also always a risk of contamination of the device.^18^

There is a growing tendency, and evidence to support, use of the chimney technique in this high-risk group of patients, primarily related to the low early mortality and complication rates. Results for the multicenter PERICLES^19^ study (Prospective study for improvement of colonoscopy bowel preparation procedure by software supported visualization) in 2015, included 517 patients treated with the chimney technique, with a total of 898 chimney prostheses. The mean follow-up time was 17 months, and the survival rate in a cohort of high-risk patients was 79%, with primary patency of 94% and secondary patency of 95%. Additionally, a French study published in 2014 did not detect significant differences in short or medium term results between patients treated with the chimney technique and the fenestrated technique to repair juxtarenal AAA. Therefore, the chimney technique should be considered as a valid, off-the-shelf, and immediately available option for treating complex aneurysms in a group of high-risk patients.^19^

We therefore conclude that, in this case, ET with the technique employing an endoprosthesis with parallel stents (chimney) for a complex ruptured AAA proved to be an excellent treatment option. This was primarily due to its availability at the time of the emergency. It is a feasible and reproducible technique, in addition to being inexpensive in comparison with other endovascular techniques. Further studies are needed to assess the role of ET in treatment of ruptured AAA over the long term.
