# Supplementary material for: Immediate outcome of endovascular treatment of ruptured juxtarenal aneurysm with parallel stents
Source: J Vasc Bras. 2021 Mar 15;20:e20200120. doi: 10.1590/1677-5449.200120 (PMC8147893; doi:10.1590/1677-5449.200120)
Supplement: Translation

# Translation: Resultado imediato do tratamento endovascular com stents paralelos do aneurisma roto justarrenal

**Authors:** Claudia Guimarães Agle, César Amorim Pacheco Neves, Flávia Dórea Carneiro Abbehusen, Tainã Lemos Andrade, Filinto Marques de Cerqueira, Dejean Sampaio Amorim

**Affiliations:** 1 Centro Universitário FTC – UniFTC, Salvador, BA, Brasil.; 2 Associação Bahiana de Medicina – ABM, Salvador, BA, Brasil.; 3 Colégio Brasileiro de Radiologia – CBR, São Paulo, SP, Brasil.; 4 Fundação Bahiana de Cardiologia – FBC, Salvador, BA, Brasil.

**Keywords:** aneurisma roto, procedimentos endovasculares, aneurisma da aorta abdominal, stents, ruptura

## Abstract

A ruptura do aneurisma de aorta abdominal é um evento com alta mortalidade, e o seu tratamento nesses casos é uma emergência médica. O tratamento endovascular desses aneurismas tem se estabelecido como uma alternativa minimamente invasiva à cirurgia aberta clássica, tornando-se a opção de primeira escolha. Contudo, 20 a 50% dos pacientes portadores de aneurisma de aorta abdominal não apresentam anatomia favorável para o tratamento endovascular devido à presença de colo curto ou pelo acometimento de ramos viscerais pelo aneurisma. Relatamos um caso de uma paciente de 70 anos submetida à correção endovascular de aneurisma roto justarrenal com implante de stents paralelos para as renais (técnica de chaminé). São apresentados dados clínicos e detalhes do procedimento. O sucesso técnico foi obtido e não houve relato de complicações pós-operatórias.

## INTRODUÇÃO

O aneurisma de aorta abdominal (AAA) constitui um desafio para o cirurgião vascular, especialmente quando se estende para os vasos viscerais (aneurismas complexos). A ruptura do AAA é um evento com alta mortalidade, e a cirurgia ou o tratamento endovascular nesses casos é uma emergência médica. O risco de ruptura do aneurisma é proporcional ao seu tamanho, sendo que aneurismas medindo menos de 5,4 cm têm uma taxa de ruptura anual de aproximadamente 1%, enquanto aqueles maiores que 7,0 cm de diâmetro têm uma taxa de ruptura anual de 32,5%^1^. Esse risco é ainda quatro vezes maior em mulheres do que em homens^2^. O tratamento endovascular (TE) desses aneurismas tem se estabelecido como uma alternativa minimamente invasiva à cirurgia aberta clássica, tornando-se a opção de primeira escolha. Essa evolução é baseada principalmente nos ensaios multicêntricos randomizados EVAR-1 (*Endovascular Aneurysm Repair 1*), DREAM (*Diabetes Reduction Assessment with ramipril and rosiglitazone Medication*) e OVER (*Open versus Endovascular Repair*)^3^^-^ 6. Contudo, 20 a 50% dos pacientes portadores de AAA não apresentam anatomia favorável para o TE devido à presença de colo curto ou pelo acometimento de ramos viscerais pelo aneurisma, e sabe-se que AAAs com comprimento do colo proximal menor que 10 milímetros estão associados a um risco aumentado de reintervenção e óbito^7^. Entre as opções para esses casos, são factíveis as técnicas com endopróteses fenestradas, ramificadas, modificadas pelo cirurgião e stents paralelos. A técnica com stents paralelos (chaminé) baseia-se na colocação de stents nos vasos viscerais em paralelo ao corpo principal da endoprótese^8^. Apresentamos aqui um caso de ruptura de AAA justarrenal tratado através da técnica endovascular com implante de stents paralelos para as renais em um serviço particular na cidade de Salvador, BA.

O estudo foi aprovado pelo Comitê de Ética da Fundação Bahiana de Cardiologia (FBC) de Salvador, BA, Brasil (Parecer nº 4.341.672).

## PARTE I – SITUAÇÃO CLÍNICA

Paciente do sexo feminino, 70 anos. Tabagista, hipertensa, foi encaminhada ao serviço com dor intensa em flanco direito e pelve. Foi feita suspeita diagnóstica de nefrolitíase e solicitada tomografia com contraste. Esta identificou aneurisma fusiforme de 8 cm com sinais sugestivos de rotura (Figura 1) e justarrenal (Figuras 2 e 3).

**Figura 1:** Angiotomografia demonstrando aneurisma da aorta abdominal (AAA) roto com hematoma retroperitoneal à direita. Seta vermelha: ruptura do aneurisma (descontinuidade da parede do AAA). Seta amarela: hematoma retroperitoneal.

**Figura 2:** Angiotomografia demonstrando aneurisma da aorta abdominal justarrenal.

**Figura 3:** Reconstrução tomográfica demonstrando aneurisma da aorta abdominal justarrenal.

Diante desse quadro, algumas opções terapêuticas foram aventadas:

1- Procedimento endovascular (endoprótese fenestrada, ramificada, modificada pelo cirurgião ou técnica com stents paralelos);
2- Cirurgia aberta convencional.

## PARTE II – O QUE FOI FEITO

A paciente foi submetida à anestesia geral, e o tratamento endovascular foi realizado. Quatro acessos foram necessários para o procedimento: dissecção de artéria femoral comum bilateral e punção das artérias braquiais. Foi inserido na artéria femoral direita um introdutor 12F, na artéria femoral esquerda, um introdutor 16F e nas artérias braquiais, um introdutor 7F longo. Pela artéria femoral esquerda, foi posicionado o corpo da endoprótese (Figura 4) GORE C3^®^ (W. L. Gore & Associates, Inc., Delaware, EUA) (26 x 14 x 18) próximo à artéria mesentérica superior. Pelas artérias braquiais, foram cateterizadas as artérias renais e posicionados os stents autoexpansíveis recobertos VIABAHN 6 x 50 (Figura 5). O corpo da endoprótese foi liberado até a perna contralateral, e, em seguida, foram liberados os stents das artérias renais (Figura 6). Após isso, pela artéria femoral direita, a extensão contralateral da endoprótese (16 x 14 x 12) foi implantada. Foi realizada aortografia de controle, demostrando endoprótese sem vazamentos e com perviedade de artérias renais (Figuras 7 e 8). Não foi utilizado balão de acomodação no seguimento proximal da endoprótese. A paciente permaneceu na unidade de terapia intensiva (UTI) por 24 horas, tendo alta hospitalar após 48 horas. Realizou-se tomografia de controle com 30 dias, que demonstrou endoprótese e stents de artérias renais bem-posicionados e sem sinais de fratura (Figura 9).

**Figura 4:** Corpo da endoprótese posicionada na aorta proximal, com cateterização seletiva da renal esquerda.

**Figura 5:** Stents autoexpansíveis VIABAHN posicionados nas artérias renais.

**Figura 6:** Corpo da endoprótese e stents autoexpansíveis das renais liberados.

**Figura 7:** Aortografia de controle demonstrando seguimento proximal da endoprótese sem vazamentos e stents de artérias renais bem-posicionados.

**Figura 8:** Aortografia de controle demonstrando seguimento distal da endoprótese sem vazamentos.

**Figura 9:** Angiotomografia de controle com 30 dias mostrando endoprótese e stents de artérias renais bem-posicionados, sem fraturas e sem vazamentos.

## DISCUSSÃO

O tempo de intervenção para o AAA roto afeta diretamente os resultados. Estes pacientes devem ter seu diagnóstico identificado o mais precocemente possível, com rápido encaminhamento a uma unidade hospitalar cuja estrutura permita o tratamento adequado da patologia. Segundo a diretriz publicada em 2018 pela *Society for Vascular Surgery* (SVS), a meta de intervalo porta-balão deve ser menor que 90 minutos, com o tempo zero sendo definido como o tempo do primeiro contato médico, e a intervenção definida a partir do acesso arterial inicial e colocação de um balão de oclusão aórtica. Apesar da indicação, cumprir o atendimento nesse curto espaço de tempo ainda é desafiador. Em 2004, o Registro Nacional de Dados Cardiovasculares dos Estados Unidos informou que, para pacientes que necessitaram de transferência inter-hospitalar, apenas 8,6% tinham cumprido o tempo porta-balão menor que 90 minutos, com um tempo médio de 152 minutos^9^.

Atualmente, o TE do AAA roto é realizado nos pacientes estáveis, já que é necessária a realização prévia de uma angiotomografia que defina as medidas adequadas para esse tipo de conduta. Contudo, existem protocolos nos centros de referência com grande demanda de casos que tendem a se beneficiar do uso do REBOA (oclusão ressuscitativa por meio de balão endovascular da aorta), o que amplia e possibilita o TE para pacientes instáveis^10^. Vale lembrar que essa conduta não é consenso e ainda é pouco discutida no Brasil, não tendo sido realizada no nosso caso.

Nos Estados Unidos, uma análise das tendências confirmou que o EVAR está sendo cada vez mais utilizado para o tratamento dos AAA rotos, com diminuição da mortalidade associada. Também demonstrou resultados superiores quando o EVAR para aneurisma roto é realizado em hospitais de ensino e centros de alto volume. Se anatomicamente favorável, a sua opção pelo TE em detrimento da cirurgia aberta foi classificada como forte nível de recomendação (nível 1)^11^. No Brasil, segundo a Diretriz Brasileira para o tratamento do AAA da Comissão Nacional de Incorporação de Tecnologias no Sistema Único de Saúde (CONITEC), os AAAs rotos estáveis, com anatomia favorável ao tratamento por EVAR (confirmada pela tomografia computadorizada), devem ser tratados em caráter emergencial por cirurgia aberta ou EVAR de acordo com a experiência e disponibilidade da equipe cirúrgica e material^2^.

O estudo multicêntrico e randomizado IMPROVE (*Immediate Management of Patients with Ruptured Aneurysm: Open Versus Endovascular Repair*) demonstrou uma mortalidade em 30 dias semelhante para o reparo aberto (37,4%) e endovascular (EVAR) (35,4%). No entanto, o EVAR teve menor tempo de permanência hospitalar. Há também uma tendência de melhores resultados em mulheres^12^.

As endopróteses constituem uma parcela significativa dos custos do EVAR (34-52%), mas esses custos são compensados pelo menor tempo de internação, o que difere do reparo aberto. Porém, a longo prazo, não são observadas diferenças significativas com relação ao custo, devido à necessidade de acompanhamento com imagens e reintervenções após o EVAR. Os pacientes submetidos ao EVAR geralmente têm melhor qualidade de vida relacionada à saúde nos primeiros 12 meses, embora estudos não tenham mostrado diferença significativa além do primeiro ano^13^.

Entre os benefícios do procedimento endovascular sobre o reparo aberto, estão a invasividade reduzida, evitando uma laparotomia, o controle vascular sob anestesia local, a estabilidade hemodinâmica, a hipotensão permissiva durante todo o procedimento e a oclusão aórtica seletiva para reduzir a hemorragia retroperitoneal. Isso pode resultar em tempo de operação mais rápido, menor perda de sangue e redução da morbidade e mortalidade cardiopulmonar perioperatória, possivelmente resultando em melhor desfecho da mortalidade em 30 dias e melhor desfecho a longo prazo^14^. Porém, é importante ter atenção com o tamanho do aneurisma corrigido, pois já foi relatado que, quanto maior o diâmetro do AAA, observado principalmente em casos rompidos, maior é a associação com as taxas de reintervenção^15^.

A técnica com stents paralelos consiste em colocar stents revestidos nas artérias viscerais, em paralelo ao corpo da endoprótese da aorta. É chamada de periscópio quando é colocado em sentido retrógrado ao fluxo sanguíneo e chamada de chaminé, *snorkel* e sanduíche quando colocado em sentido anterógrado ao fluxo. Normalmente, para o TE do AAA, é utilizado um *oversize* de 10 a 15% na endoprótese. Contudo, na técnica de chaminé, se faz necessário um *oversize* maior (30%), para evitar possíveis vazamentos e goteiras (“*gutters*”). Também é importante um *overlap* (comprimento) de pelo menos 5 cm de stent paralelo, terminando 1 cm acima da endoprótese da aorta^16^. Não se deve usar balonamento de acomodação na aorta devido ao risco de compressão dos stents paralelos das viscerais.

A escolha pela técnica de chaminé ocorreu por diversos motivos: fácil acesso ao material necessário para o TE, experiência da equipe com essa técnica e possibilidade de tratamento em curto espaço de tempo. Além disso, há uma limitação para o uso de endoprótese fenestrada, já que o seu tempo de confecção (6-8 semanas) é elevado, pois são produzidas especificamente para cada caso. Já para as endopróteses ramificadas, a necessidade de um diâmetro mínimo de 34 mm impossibilitou o seu uso. Quanto às endopróteses de prateleira, ainda são disponíveis no mercado apenas com quatro ramos ou fenestras (não se adequando ao caso, no qual era necessário cobrir apenas as renais)^17^.

Quanto às endopróteses modificadas pelo cirurgião, é necessário ter mais experiência com relação à manipulação e modificação dos dispositivos. Além disso, há sempre o risco de contaminação do material^18^.

Há uma tendência crescente e evidências para apoiar o uso da técnica de chaminé nesse grupo de alto risco de pacientes, principalmente relacionadas às baixas taxas precoces de mortalidade e complicações. Resultados do estudo multicêntrico PERICLES^19^ (*Prospective study for improvement of colonoscopy bowel preparation procedure by software supported visualization*) em 2015 mostraram 517 pacientes submetidos a técnica de chaminé, com um total de 898 próteses de chaminé. O tempo médio de seguimento foi de 17 meses, e a taxa de sobrevivência em uma coorte de alto risco de pacientes foi de 79%, com uma taxa primária de patência de 94% e uma taxa secundária de 95%. Além disso, um estudo francês publicado em 2014 não encontrou diferença significativa nos resultados de curto e médio prazo entre pacientes tratados com a técnica de chaminé e a técnica fenestrada para o AAA justarrenal. Sendo assim, a técnica de chaminé deve ser considerada válida fora da prateleira e imediatamente disponível para tratar aneurismas complexos em um grupo de pacientes de alto risco^19^.

Concluímos, então, com esse caso, que o TE com a técnica de endoprótese com stents paralelos (chaminé) no AAA roto complexo se mostrou uma excelente opção terapêutica. Isso se deve principalmente à sua disponibilidade na urgência. É uma técnica factível e reprodutível, além de ser de baixo custo quando comparada às outras técnicas endovasculares. Outros estudos para avaliar o papel do TE no tratamento do AAA roto a longo prazo são necessários.
